# Thrombectomy aspiration post-market study in acute stroke with the Q aspiration catheter: the TAPAS study

**DOI:** 10.1136/neurintsurg-2022-018649

**Published:** 2022-05-31

**Authors:** Mariano Espinosa de Rueda, Federico Ballenilla Marco, Eñaut Garmendia Lopetegui, Jose M Pumar, Joaquin Zamarro, Blanca García-Villalba, Jose Díaz-Pérez, Antonio Mosqueira, Alex Lüttich, Jose-Angel Larrea, Guillermo Parrilla

**Affiliations:** 1 Department of Interventional Neuroradiology, Hospital Clínico Universitario Virgen de la Arrixaca, Murcia, Spain; 2 Department of Interventional Neuroradiology, Hospital Universitario 12 de Octubre, Madrid, Spain; 3 Department of Interventional Neuroradiology, Hospital Universitario de Donostia, San Sebastián, Spain; 4 Department of Interventional Neuroradiology, Hospital Clínico Universitario de Santiago de Compostela, Santiago de Compostela, Spain

**Keywords:** Stroke, Thrombectomy, Catheter, Device

## Abstract

**Background:**

The Q Aspiration Catheter (MIVI Neuro) has demonstrated greater aspiration flow rates and ingestion forces compared with conventional catheters in vitro. The safety and performance of the Q Catheter was assessed using a direct aspiration first pass technique in patients with acute ischemic stroke at four neurointerventional centers in Spain.

**Methods:**

We included adult patients who underwent mechanical thrombectomy between March 2019 and March 2020 using the Q Catheter as first-line therapy. Performance endpoints included final successful revascularization of the target vessel (defined as modified thrombolysis in cerebral infarction (mTICI) grade 2B/3), first pass revascularization, and overall Q Catheter revascularization. Safety endpoints were symptomatic intracranial hemorrhage (sICH), embolization to new territory (ENT), and procedural complications. Modified Rankin Scale (mRS) score and all-cause mortality were also assessed.

**Results:**

Forty-five subjects were enrolled. The Q Catheter successfully navigated to the lesion in 95.5% (43/45) of patients. Final successful mTICI 2B/3 revascularization was achieved in 93.3% (42/45), first pass mTICI 2B/3 revascularization with the Q Catheter was 55.3% (21/38), and overall with Q Catheter mTICI 2B/3 revascularization was 65.8% (25/38). Favorable clinical outcome of mRS 0–2 was achieved in 55.6% (25/45). There were no cases of ENT. sICH and mortality rates were 2.2% (1/45) and 13.3% (6/45), respectively.

**Conclusion:**

In this multicenter, observational study, the Q Aspiration Catheter used as first-line therapy demonstrated a good and safe profile in terms of navigation, revascularization, and safety in patients with acute ischemic stroke.

## Introduction

Mechanical thrombectomy (MT) is the standard of care for patients experiencing acute ischemic stroke (AIS) with a large vessel occlusion (LVO). Since the beginning of the technique, different devices have been approved for use in the removal of thrombus from the brain vasculature, including stent retrievers^1^ and aspiration catheters.^2 3^

MIVI Neuroscience (Minnesota, USA) has developed a novel catheter called the MIVI Q Aspiration Catheter. The Q Catheter is a two-part telescoping system composed of a short and flexible distal catheter section mounted on a proximal 0.020 inch control wire, used in combination with an 8F guide catheter. This unique design of the Q Catheter has been shown in bench studies to offer hemodynamic advantages over standard, fixed-diameter thrombectomy catheters.^4^

This multi-center, observational, post-market study is the first reported clinical experience with the Q Aspiration Catheter. The study was designed to provide initial safety and performance data on the device used as first-line therapy during MT for AIS at four large hospitals in Spain.

## Materials and methods

### Patient selection

Four neurointerventional centers in Spain participated in this observational study, which was approved by the correspondent lead and/or local ethics committees (CEIC Hospital Virgen de la Arrixaca No 2019-3-15-HCUVA). We retrospectively studied prospectively recorded data of patients with AIS treated by a study investigator with MT using the Q Catheter as first-line therapy between March 2019 and March 2020. The use of the Q Catheter was at the neurointerventionist’s discretion at the time of the thrombectomy. Patients who met the inclusion criteria, or family members where applicable, were asked to provide written informed consent to participate in the study. Data were collected at baseline after obtaining consent, and at follow-up visits.

The inclusion criteria for the study were as follows: age 18–85 years; procedure initiated within 8 hours of symptom onset or last time known well (LKW); LVO of the anterior circulation (intracranial internal carotid artery (IICA) and M1 or M2 segments of the middle cerebral artery (MCA)) or posterior circulation (intracranial vertebral or basilar artery (VBA)); Alberta Stroke Protocol Program Early CT Score (ASPECTS) 6–10; and use of the Q Catheter as first-line treatment according to the instructions for use. The exclusion criteria were occlusion in multiple vascular territories, extracranial or tandem occlusion, evidence of target artery dissection, or fresh or recent hemorrhage. Thrombolytic therapy was initiated as per standard protocol according to current guidelines.

### Q Aspiration Catheter

The Q Aspiration Catheter consists of a short single lumen, variable stiffness shaft (25–43 cm) with radiopaque markers on the distal and proximal ends of the catheter portion for angiographic visualization. The catheter shaft has a hydrophilic coating to reduce friction during use. The proximal portion of the Q Catheter is a 105 cm, 0.020 inch stainless steel wire that is used to advance and retract the catheter within an 8F guide catheter. The Q Catheter is available in four sizes (Q3, Q4, Q5 and Q6, corresponding to 3F, 4F, 5F and 6F diameters, respectively) to accommodate the target artery diameter. The proximal outer diameter of the catheter section flares to create a sliding seal between it and the 8F guide catheter, as shown in online supplemental figure 1. This design augments aspiration flow rates by up to 240%.^4^ When performing the thrombectomy, a vacuum is attached to the 8F guide catheter via the rotating hemostasis valve (RHV). Utilizing the 8F guide catheter to transmit the vacuum to the tip of the Q Catheter in this way provides a significantly larger diameter lumen to maximize aspiration. This combination allows greater fluid flow and larger thromboemboli ingestion forces that may enable faster and more complete clot removal.

10.1136/neurintsurg-2022-018649.supp1 Supplementary data

### Procedural technique

Inclusion in the study was based on the intent to use the Q Catheter as first-line therapy during MT with a direct aspiration first pass technique (ADAPT).^5^ Procedural anesthetic technique (general anesthesia or conscious sedation) depended on each institution criteria. Via transfemoral approach an 8F guide catheter (Super 90 or Neuron Max 0.088) was positioned in the distal portion of the cervical internal carotid artery (or the vertebral artery in posterior circulation cases) using the standard technique. The Q catheter can be introduced in the 8F guide catheter either before entering the patient or once the 8F is already positioned in the internal carotid artery or the vertebral artery. The Q Catheter is advanced to the occlusion by pushing its wire portion and with the help of a micro-wire and/or micro-catheter, which are pre-loaded in the lumen of the Q to facilitate navigation. Once the system is near the occlusion, the Q is advanced over the microcatheter to impact the thrombus. Aspiration tubing is then connected to the guide catheter RHV, the microcatheter/microwire is removed, and aspiration of the thrombus is initiated. The Q Catheter is completely removed from the patient’s body while continuous aspiration is maintained in the guide catheter

In the event that the Q Catheter failed to navigate to the lesion, an anchoring technique with a stent retriever was permitted.^6 7^ This technique places a stent retriever at or beyond the thrombus and provides support for advancing the aspiration catheter to the thrombus. Once the thrombus is reached by the Q Catheter, thrombectomy is performed using a combined technique with aspiration and stent retriever.

If the Q Catheter failed to recanalize the vessel after the first attempt, the physician decided whether to continue using the same technique in successive passes or switch to other commercially available devices.

### Study endpoints

The primary performance endpoint was successful final revascularization of the target vessel (defined as modified thrombolysis in cerebral infarction (mTICI) grade 2B/3), and the primary safety endpoint was post-procedural symptomatic intracranial hemorrhage (sICH).^8^

The secondary endpoints were Q Catheter first-pass revascularization (mTICI 2B/3 and 2C/3), Q Catheter overall revascularization (mTICI 2B/3 and 2C/3), embolization to new territory (ENT), procedural complications, functional outcome (defined as 3-month modified Rankin Scale (mRS) score), and all-cause mortality.

An independent neuroradiologist blinded for the study reviewed all procedural angiograms and post-procedure neuroimaging for safety. Patients were followed during hospitalization and contacted for clinical follow-up and mRS evaluation at three or more months after the procedure.

### Statistics

All endpoints were analyzed descriptively using SAS Version 9.4 (Cary, NC, USA). Continuous parameters were summarized by number of evaluable observations, mean, SD, median, minimum, and maximum. Categorical data were described by frequency counts and percentages, with 95% exact binomial confidence intervals added where applicable.

## Results

### Study population and procedural data

A total of 86 thrombectomies were performed by study investigators using the Q Catheter as first-line ADAPT therapy during the study period. Of those, 58 patients were eligible and 45 consented to participate (final population of the study). Thirteen patients could not be contacted or declined to participate. Reasons for the exclusion of the 28 patients who failed to meet the entry criteria are shown in online supplemental figure 2.

Baseline and stroke characteristics are detailed in table 1. The mean age was 72.4 years, and 53.3% of the patients were male, with a mean National institute of Health Stroke Scale of 14.2 on presentation. A total of 54.8% presented an ASPECTS ranging from 6 to 9, and 45.2% presented an ASPECTS of 10. Previous medical history was positive for hypertension, hypercholesterolemia and atrial fibrillation in 71.1%, 59.1%, and 43.2% of patients, respectively. Online supplemental table 1 shows the comparison between the baseline characteristics of this population and those of other large bore catheter studies.

**Table 1:** Baseline and stroke characteristics

Characteristic	Frequency
Age (years)	72.4±8.8 (range 49–85)
Male sex	53.3% (24/45)
Pre-stroke mRS	
0	57.8% (26/45)
1	26.7% (12/45)
2	11.1% (5/45)
3	4.4% (2/45)
NIHSS score	14.2±6.7
Smoker	47.7% (21/44)
Diabetes mellitus	28.9% (13/45)
Hypertension	71.1% (32/45)
Hypercholesterolemia	59.1% (26/44)
Cerebrovascular disease	15.6% (7/45)
Atrial fibrillation	43.2% (19/44)
Myocardial infarction	8.9% (4/45)
Heart valve replacement	6.7% (3/45)
Pulmonary disease	17.8% (8/45)
ASPECTS score	
6	9.5% (4/42)
7	14.3% (6/42)
8	14.3% (6/42)
9	16.7% (7/42)
10	45.2% (19/42)
Location of occlusion	
Left hemisphere	44.4% (20/45)
Right hemisphere	48.9% (22/45)
VBA circulation	6.7% (3/45)

Data are mean±SD (range), or % (n/N).

ASPECTS, Alberta Stroke Protocol Programme Early CT Score; mRS, modified Rankin Scale; NIHSS, National institute of Health Stroke Scale; VBA, vertebro basilar artery.

Procedural data are reported in table 2. Intravenous tissue plasminogen activator (IV-tPA) was administered in 46.7% of the subjects, and 68.9% of the procedures were performed under general anesthesia (see data by center in online supplemental table 2). Super-90 was used as the guide catheter in 64.4% of patients. The main occlusion location was the M1 segment of the MCA (55.5%), followed by the IICA (22.2%). A total of 57 Q Catheters were used to perform the thrombectomies in the 45 patients, with the Q6 and Q5 sizes used most frequently (30 and 19 catheters, respectively). The median number of revascularization attempts was 2.0, with a median procedure time of 40 min. The predominant final mTICI score was 3 (66.7%), followed by 2B (20%) and 2C (6.7%).

**Table 2:** Procedural data

Characteristic	Frequency (n=45)
IV rTPA administered	46.7% (21)
Sedation	
Conscious sedation	31.1% (14)
General anesthesia	68.9% (31)
Site of occlusion	
IICA	22.2% (10)
M1	55.5% (25)
M2	15.6% (7)
VBA	6.7% (3)
Time from onset/LKW to procedure start (min)	
Mean±SD	274.2±144.1
Guide catheter	
MIVI Neuroscience Super 90	64.4% (29)
Penumbra Neuron Max	35.6% (16)
Q Catheter sizes used	
Q3	4
Q4	4
Q5	19
Q6	30
Number of revascularization attempts per patient (any device)	
Mean±SD	2.7±2.1
Median	2.0
Min, max	1.0, 9.0
Procedure time (min)*	
Median	40
Min, max	5.0, 229.0
Final mTICI score	
0	2.2% (1)
2A	4.4% (2)
2B	20% (9)
2C	6.7% (3)
3	66.7% (30)

Data are mean±SD; min, max; median; or % (n/N).

*Groin puncture to final revascularization, in minutes.

IICA, intracranial internal carotid artery; IV rTPA, intravenous recombinant tissue plasminogen activator; LKW, last known well; mTICI, modified thrombolysis in cerebral infarction; VBA, vertebro basilar artery.

### Navigation, revascularization and safety outcomes

As shown in table 3, the Q Catheter was successfully positioned inside the occlusion in 95.5% (43/45) of cases. In 84.4% (38/45), the Q Catheter successfully navigated to the target, and in five cases (11.1%) the assistance of a stent retriever for distal anchorage was needed. In these five cases a combined thrombectomy technique was used of stent retriever and aspiration. In two patients, the Q Catheter failed to reach the target occlusion, necessitating the use of other commercially available devices.

**Table 3:** Navigation, revascularization, and safety data

Characteristic	Frequency
Navigation	
Q Catheter alone	84.4% (38/45)
Q Catheter+stent retriever anchoring	11.1% (5/45)
Failed	4.5% (2/45)
Revascularization	
First pass Q Catheter	
mTICI 2B-3	55.3% (21/38)
mTICI 2 C-3	39.5% (15/38)
Overall Q Catheter*	
mTICI 2B-3	65.8% (25/38)
mTICI 2 C-3	47.4% (18/38)
Final result	
mTICI 2B-3	93.3% (42/45)
mTICI 2 C-3	73.3% (33/45)
Intracranial hemorrhage	
Asymptomatic	35.6% (16/45)
Symptomatic	2.2% (1/45)
Procedural complications	
Reversible angiographic vasospasm	9.3% (4/45)
Vessel dissection	2.2% (1/45)
Groin hematoma	2.2% (1/45)
Clinical outcome (follow-up mRS)	
0–2	55.6% (25/45)
3–5	31.1% (14/45)
6	13.3% (6/45)

Data are % (n/N).

*One or more passes, one or more Q Catheters.

mRS, modified Rankin Scale; mTICI, modified thrombolysis in cerebral infarction.

First-pass revascularization mTICI 2B/3 with the Q catheter was achieved in 55.3% of patients (21/38) and mTICI 2C/3 in 39.5% (15/38). Overall Q Catheter mTICI 2B/3 revascularization after one or more passes was 65.8% (25/38) and for mTICI 2C/3 it was 47.4% (18/38). The rate of final mTICI 2B/3 revascularization was 93.3% (42/45) and for mTICI 2C/3 it was 73.3% (33/45). In cases where the recanalization with the Q Catheter was not satisfactory, additional devices used included aspiration catheters, stent retrievers, and/or both: Penumbra ACE68 and 3MAX, Medtronic Solitaire, Stryker Trevo, Cerenovus Embotrap, and Balt Catch (online supplemental table 3). Three patients also required angioplasty and permanent stent placement for intracranial stenosis.

Procedural complications included one internal carotid artery dissection (2.2%), four angiographic vasospasms (9.3%), and one procedural groin hematoma (2.2%). The arterial dissection was caused by the positioning of the guide catheter in the cervical segment of the ICA and was treated with the deployment of a stent. All cases of local angiographic vasospasms were related to the advance of the guiding catheter and resolved spontaneously. There were no cases of ENT.

The rate of sICH was 2.2%, corresponding to one patient who suffered a massive hemorrhagic transformation 2 days after thrombectomy and eventually died. The all-cause mortality rate was 13.3% (6/45), and among the surviving patients, the rate of follow-up mRS 0–2 was 55.6% (25/45), and for mRS 3–5 it was 31.1% (14/45).

## Discussion

In this multicenter clinical study on the novel MIVI Q Aspiration Catheter, we found that the device was technically successful in navigating to the clot in most cases, with rates of first pass and successful recanalization that are preliminarily comparable to other large bore aspiration catheters (table 4). While previous studies have shown the good performance of the Q Catheter when used in combination with SR,^9^ these are the first results to demonstrate the feasibility of its use as first-line therapy for AIS.

**Table 4:** Comparison of safety and effectiveness with large bore aspiration catheters

Reference	Patients/device	mTICI 2B-3 (final/only aspiration)	Safety (sICH/ENT)	mRS 0–2 90 days	Mortality
Mohlenbruch (2019)^10^	85/SOFIA PLUS (Microvention)	96.5%/64.7%	4.7%/4.7%	49.4%	20%
De Marini (2019)^11^	41/ARC (Medtronic)	98%/85%	2%/2.5%	49%	10%
Bretzner (2019)^12^	60/AXS Catalyst (Stryker)	86.7%/83.3%	5%/NR	48.3%	21.7%
Marnat (2019)^13^	296/SOFIA 5–6F (Microvention)	86.1%/NR	6.2%/NR	43%	22.9%
Schramm (2019)^15^	204/ACE64/68 (Penumbra)	93.1%/70.6%	2.9%/1.5%	61%	7.5%
Raymond (2020)^16^	47/REACT 68 (Medtronic)	95.7%/53.2%	4.3%/NR	23.5%	NR
Bilgin (2021)^14^	148/SOFIA 6F (Microventrion)	89.1%/69.2%	5.4%/5.4%	49.3%	14.1%
**TAPAS**	**45/MIVI Q**	**93.3 %/73.3%**	**2.2%/0%**	**55.6%**	**13.3%**

ENT, embolism new territory; NR, not reported; sICH, symptomatic intracranial hemorrhage.

The Q Catheter design maximizes lumen size to increase aspiration and ingestion forces flow without increasing the catheter tip diameter. The proximal two thirds of the catheter have been replaced by a stainless-steel control wire and the distal third is a flexible catheter with a proximal end flare design to match the inner diameter of the guide catheter and permit a continuous vacuum from the distal tip of the Q Catheter through the 8F guide catheter. During retraction of the Q Catheter into the guide catheter, the aspiration flow rate at the tip of the Q is maximized. Once the Q Catheter proximal flare end reaches the RHV on the guide, aspiration is transferred completely to the guide catheter and maximized to ingest the clot. This design has demonstrated a substantial increase in aspirated flow rate and suction force due to an increased effective diameter compared with standard catheters.^4^ A single suction line provides suction to both the Q Catheter and the guide catheter, reducing the need for separate suction lines to avoid clot loss as the aspiration catheter is drawn into the guide catheter.

The population of this study was similar to those of other large bore catheter studies (table 1 and online supplemental table 1): patients with AIS in their early 70s (mean 72.4 years), with similar gender distribution (53.3% male), and high blood pressure as main cardiovascular risk factor (71.1%). The most frequent occlusion sites were the M1 segment of the MCA (55.5%) and the ICA (22.2%).

In terms of navigation, the results of this study show that the soft and flexible design of the Q Catheter resulted in good and safe navigation to the target occlusion. The different available sizes (from 3 to 6F diameters) permitted lesions located in proximal or distal occlusion sites to be reached, from the intracranial ICA to M2 segment. Mohlenbruch *et al* 10 reported a successful positioning of the SOPHIA plus catheter inside the occlusion without the help of a SR in 91% of cases, and De Marini *et al* 11 reported successful navigation in 84% of the treated patients when using only the ARC catheter, similar to the 84% obtained by the Q Catheter in our study. Interestingly, the use of a SR as distal anchorage^6 7^ permitted the advance of the Q Catheter to reach the thrombus in five out of seven cases of difficult navigation, similar to what was described with the ARC in five out of six cases.^11^

First-pass mTICI 2B/3 rates for other large bore catheters like ARC,^11^ AXS Catalyst,^12^ and SOPHIA^10 13 14^ range from 24% to 60%, which are similar to our first-pass results (55.3%). Interestingly, overall Q Catheter results reached mTICI 2B/3 reperfusion rates superior to 65% in our series of patients. These data seem to indicate that multiple passes and/or the combination of different size Q Catheters may significantly increase the rate of successful revascularization without the need for other concomitant thrombectomy devices.

The final successful revascularization mTICI 2B/3 rate in this series is 93.3%, comparable to the range of 84%–98% obtained by the ARC,^11^ AXS Catalyst,^12^ ACE 64/68,^15^ React 68,^16^ and SOPHIA catheters.^10 13 14^ Since final revascularization rates include the use of different thrombectomy devices and techniques, our results illustrate the compatibility and efficacy of the Q Catheter with other materials in order to achieve a reopening of the vessel in complicated or resistant occlusions.

This study also demonstrated low complication rates in a real-world population. Interestingly, there were no cases of ENT after treatment with Q Catheters, while cases were reported in 5.4% of patients for Sofia 6F,^14^ 4.7% for Sofia Plus,^10^ and 2.5% for ARC.^11^ This absence of ENT reinforces the efficacy of the Q Catheter aspiration system and its design to maximize lumen size to increase aspiration and ingestion forces flow, and might be relevant when choosing a thrombectomy device.

Only one case of sICH was reported in this series of patients (2.2%) and apparently it was not related to the use of the Q Catheter, but rather to the evolution of infarction and massive reperfusion phenomenon 2 days after thrombectomy. The mortality rate was 13.3%, similar to that reported with other aspiration catheters (10%–22.9%).^10–16^ Finally, a favorable clinical outcome at 3 months was seen in 55.6% of our patients, similar to or slightly higher than the rates observed in populations treated with other aspiration catheters (23%–61%).^10–17^

## Limitations

Despite having favorable results and similar to other studies, this study is limited by its single-arm design and small patient population; larger controlled studies are necessary to confirm treatment effects in comparison to other thrombectomy devices. Furthermore, the study population combines patients with anterior and posterior circulation occlusions, where there are clinical-anatomical differences that may impact traceability of catheters, reperfusion rates and outcomes. Since this was an observational trial, results may be influenced by differences in treatment protocols between centers and operators.

## Conclusion

This multicenter study for stroke treatment in a small real-world population demonstrates good safety and effectiveness data for the MIVI Q Aspiration Catheter used as first-line therapy, with results comparable to similar studies of other commercially available aspiration catheters.

10.1136/neurintsurg-2022-018649.supp2 Supplementary data

## Data Availability

Data are available upon reasonable request.
