# Supplementary material for: Thrombectomy aspiration post-market study in acute stroke with the Q aspiration catheter: the TAPAS study
Source: J Neurointerv Surg. 2022 May 31;15(7):674–8. doi: 10.1136/neurintsurg-2022-018649 (PMC10314076; doi:10.1136/neurintsurg-2022-018649)

SUPPLEMENTAL MATERIAL

Thrombectomy Aspiration Post-Market Study in Acute Stroke with the Q Aspiration Catheter: The TAPAS Study

SUPPLEMENTAL TABLES

Supplemental Table 1. Comparison between baseline characteristics of TAPAS population and other populations of large bore catheter studies.

	BRETZNER 2019	MARNAT 2019	BILGIN 2021	TAPAS	t	FD	Sig (bilateral)
**AGE**	68	69,5	63,8	72,4	-1,553	2	0,261
**MEN**	42	50,7	54,7	53,3	-0,556	2	0,634
**HYPERTENSION**	62	54,9	67,5	71,1	-1,321	2	0,317
**DIABETES**	12	17,4	24,3	28,9	-1,545	2	0,262
**DYSLIPEMIA**	38	32,7		59,1	-5,174	1	0,122
**TOBACCO**	27	25,7		47,7	-18,964	1	0,034
**ADMISSION NIHSS**	17	16	14	14,2	0,832	2	0,493
**M1-OCCLUSION**	61,7	41,9		55,5	-0,216	1	0,865
**M2-OCCLUSION**	11,7	13,5		15,6	-1,925	1	0,305
**ICA-OCCLUSION**	16,7	18,2	31,7	22,2	0,000	2	1,000
**BASILAR-OCCLUSION**	10	13,2	10,8	6,7	2,409	2	0,138

Supplemental Table 2. Procedural anesthesia details by center.

SITE	POPULATION	GENERAL ANESTHESIA	CONSCIOUS SEDATION
**Center 1**	19	19	0
**Center 2**	14	2	12
**Center 3**	7	5	2
**Center 4**	5	5	0
**TOTAL**	45	31 (68.9%)	14 (31.1%)

Supplemental Table 3. Other devices used (cases in which navigation could not be achieved by the combination of Q Catheter and SR, and/or cases in which initial recanalization was not considered satisfactory). Several devices could be used in the same patient if considered necessary.

**DEVICES**	**N**
ACE 68	5
3 MAX	3
Solitaire	4
Catch	4
Embotrap	2
Trevo	3
Intracranial stent	3

SUPPLEMENTAL FIGURES

Supplemental Figure 1: Q Catheter design, shown with and without 8F guide catheter.

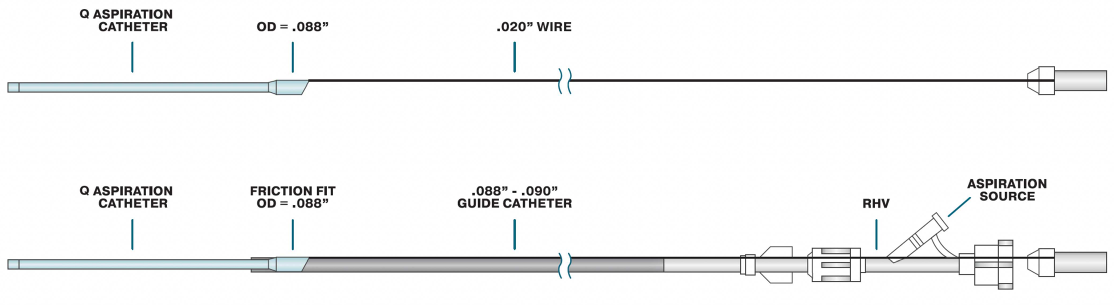

Supplemental Figure 2: Q Catheter flow chart of study participants.

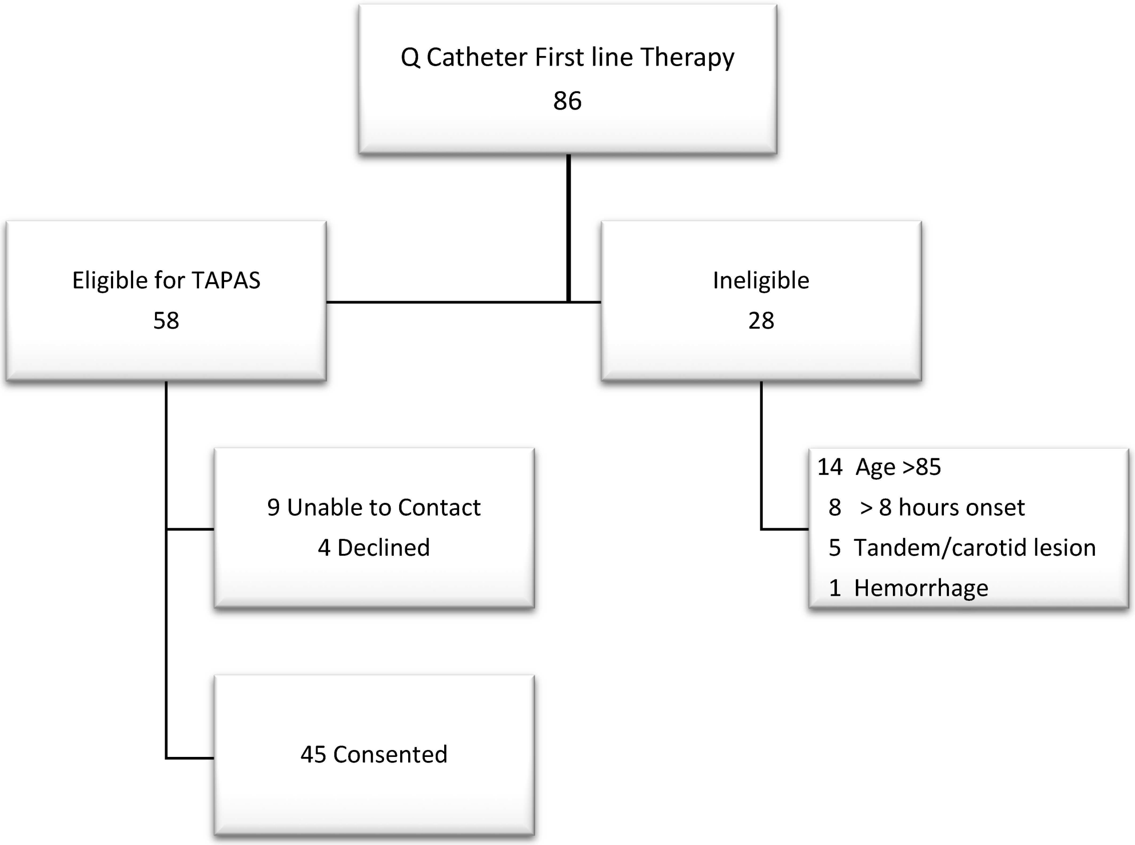

Supplement: Supplementary data [file neurintsurg-2022-018649supp001.pdf]
